# Disease-associated oligodendrocyte signatures are spatiotemporally dysregulated in spinocerebellar ataxia type 3

**DOI:** 10.3389/fnins.2023.1118429

**Published:** 2023-02-15

**Authors:** Kristen H. Schuster, Danielle M. DiFranco, Alexandra F. Putka, Juan P. Mato, Sabrina I. Jarrah, Nicholas R. Stec, Vikram O. Sundararajan, Hayley S. McLoughlin

**Affiliations:** ^1^Department of Neurology, University of Michigan, Ann Arbor, MI, United States; ^2^Neuroscience Graduate Program, University of Michigan, Ann Arbor, MI, United States

**Keywords:** Machado-Joseph disease, polyglutamine (polyQ) diseases, myelination, MJD, SCA3

## Abstract

Spinocerebellar ataxia type 3 (SCA3) is a neurodegenerative disease caused by a CAG repeat expansion in the *ATXN3* gene. Though the ATXN3 protein is expressed ubiquitously throughout the CNS, regional pathology in SCA3 patients is observed within select neuronal populations and more recently within oligodendrocyte-rich white matter tracts. We have previously recapitulated these white matter abnormalities in an overexpression mouse model of SCA3 and demonstrated that oligodendrocyte maturation impairments are one of the earliest and most progressive changes in SCA3 pathogenesis. Disease-associated oligodendrocyte signatures have recently emerged as significant contributors to several other neurodegenerative diseases, including Alzheimer’s disease, Huntington’s disease, and Parkinson’s disease, but their role in regional vulnerability and disease progression remains unexplored. Here, we are the first to comparatively assess myelination in human tissue in a region-dependent manner. Translating these findings to SCA3 mouse models of disease, we confirmed endogenous expression of mutant *Atxn3* leads to regional transcriptional dysregulation of oligodendrocyte maturation markers in Knock-In models of SCA3. We then investigated the spatiotemporal progression of mature oligodendrocyte transcriptional dysregulation in an overexpression SCA3 mouse model and how it relates to the onset of motor impairment. We further determined that regional reduction in mature oligodendrocyte cell counts in SCA3 mice over time parallels the onset and progression of brain atrophy in SCA3 patients. This work emphasizes the prospective contributions of disease-associated oligodendrocyte signatures to regional vulnerability and could inform timepoints and target regions imperative for biomarker assessment and therapeutic intervention in several neurodegenerative diseases.

## 1. Introduction

Spinocerebellar ataxia type 3 (SCA3), also known as Machado-Joseph disease, is the most common dominantly inherited ataxia in the world, yet there are currently no therapies for this fatal disease (McLoughlin et al., 2020). SCA3 is part of the polyglutamine (polyQ) expansion family of diseases and is characterized by a trinucleotide CAG repeat expansion mutation in the disease gene, *ATXN3*, that encodes a polyQ repeat in the mutant protein (Kawaguchi et al., 1994). A CAG repeat size of between 12 and 44 is typical in healthy individuals, although in SCA3 patients, the CAG expansion will reach 56–87 repeats on at least one *ATXN3* allele (Maciel et al., 1995; Durr et al., 1996). ATXN3 normally functions as a deubiquitinase (DUB) and is localized in both the nucleus and cytoplasm, however, a hallmark of disease is nuclear accumulation of ATXN3 (Paulson et al., 1997). Importantly, ATXN3 is ubiquitously expressed in all cell types throughout the human and mouse central nervous systems (CNS) (Zhang et al., 2014), yet SCA3 disease pathogenesis progresses in a spatiotemporal pattern. Widespread neuronal loss, white matter loss, and gliosis in patients are primarily observed in the deep cerebellar nuclei (DCN), pontine nuclei, spinocerebellar tract, spinal cord, and globus pallidus early in disease while other regions, such as the cortex, remain unaffected until late-stage disease (Riess et al., 2008; Rüb et al., 2008; Rub et al., 2013; Kang et al., 2014; Park et al., 2020; Wan et al., 2020). It remains unknown what underlies this regional vulnerability in SCA3 disease.

Recent reports from our lab begin to characterize disease-associated oligodendrocyte signatures in a transgenic overexpression mouse model of SCA3 (Schuster et al., 2022b,c). Through longitudinal RNA sequencing analysis, with transcriptional, biochemical, and histological confirmation, we demonstrated early and progressive dysregulation of oligodendrocyte maturation in SCA3 mice (Schuster et al., 2022c). In mid-stage SCA3 mice, we found that several myelin-rich white matter regions, including the pons, DCN, and corticospinal tract (CST), displayed a lack of mature oligodendrocytes relative to wildtype littermates at 16 weeks of age. However, in another prominent white matter tract, the corpus callosum, mature oligodendrocyte cell counts remained unaffected, although ATXN3 nuclear accumulation was still present. Moreover, we showed that mature oligodendrocyte counts correspond to the thickness of the myelin sheath, with thinner myelin sheaths in the oligodendrocyte-deprived CST and normal myelin thickness in the corpus callosum (Schuster et al., 2022b). Such work emphasizes that this neurodegenerative disease is not purely attributable to neurons, as glial cells are emerging as a key player in SCA3 pathology.

Disease-associated oligodendrocyte signatures have been described broadly throughout both polygenic and monogenic neurodegenerative diseases, including SCA3 (Ramani et al., 2017; Haas et al., 2021), other polyQ diseases such as Huntington’s disease (HD) (Yamada et al., 2001; Huang et al., 2015; Ferrari Bardile et al., 2019), and more common neurodegenerative diseases, including Alzheimer’s disease (AD), Parkinson’s disease (PD), and Amyotrophic Lateral Sclerosis (ALS) (Philips et al., 2013; Ferrari Bardile et al., 2019; Errea and Rodriguez-Oroz, 2021; Kenigsbuch et al., 2022). In both AD and SCA3, oligodendrocyte dysfunction in animal models recapitulates white matter abnormalities seen in human patients (Roher et al., 2002; Wu et al., 2017; Inada et al., 2020; Park et al., 2020; Faber et al., 2021; Haas et al., 2021; Kenigsbuch et al., 2022; Schuster et al., 2022c). In fact, non-invasive imaging of SCA3 patients revealed white matter loss to be an early feature of disease (Joers et al., 2018; Rezende et al., 2018; McLoughlin et al., 2020). The ubiquity of myelination impairments across mouse models, and more importantly, in human patients, demonstrates the significant contribution of oligodendrocytes to disease pathogenesis. However, previous studies in animal models of disease investigated relatively few timepoints and often assessed whole brain expression, causing difficulty in determining the spatiotemporal onset of oligodendrocyte dysfunction, and how it corresponds with SCA3 disease progression in vulnerable and non-vulnerable brain regions.

Because SCA3 is a monogenic neurodegenerative disease, we can employ SCA3 mouse models as a paradigmatic spatiotemporal study of disease-associated oligodendrocyte signatures. Guided by the results from these studies, we can test targeted hypotheses in other disease-relevant models to understand oligodendrocyte signatures in polygenic neurodegenerative diseases, such as AD, PD, and ALS. As a step toward exploring oligodendrocyte signatures in neurodegenerative diseases, we aim to further evaluate the spatiotemporal connections between oligodendrocyte dysfunction and disease pathogenesis in SCA3. We first highlight the relevance of regional oligodendrocyte vulnerability *via* myelin staining and MBP protein quantification in SCA3 patient post-mortem tissue. Then, using multiple mouse models of SCA3, we evaluate the corresponding regional and temporal characteristics of oligodendrocyte maturation impairments in disease. Specifically, we utilize three mouse models of SCA3: (1) the Q82 Knock-In (KIQ82) model that contains 82 CAG repeats in the mouse *Atxn3* gene to explore endogenous expression of the disease protein with a repeat size relevant to SCA3 patients (Ramani et al., 2015, 2017); (2) the recently generated Q300 Knock-In model (KIQ300) with 300 CAG repeats in the *Atxn3* gene to investigate the effects of hyperexpanded polyQ repeats expressed at endogenous levels; and (3) the YACQ84 transgenic overexpression model (denoted as “Q84”) that harbors 2 copies of the human expanded *ATXN3* gene on each chromosome and recapitulates several disease phenotypes commonly seen in SCA3 patients (Costa et al., 2013; McLoughlin et al., 2018). Further characterizing the spatiotemporal onset and progression of this oligodendrocyte signature, we assess behavioral, transcriptional, and histological characteristics of Q84 SCA3 mice. Through this work, we provide insight to disease-associated oligodendrocyte signatures, demonstrating that they correspond to the regional and temporal vulnerability in SCA3 disease pathogenesis. These findings suggest oligodendrocyte maturation dysfunction may play a pivotal role in SCA3 regional vulnerability and highlight timepoints relevant for biomarker studies and therapeutic intervention.

## 2. Materials and methods

### 2.1. Luxol fast blue

All available post-mortem human cerebellum and cortex paraffin tissue from patients with SCA3 and control subjects (cause of death was not CNS related) were acquired from the Michigan Brain Bank (Table 1). Sectioned tissues (5 μm) were deparaffinized and stained with Luxol Fast Blue MBSN (Matheson Coleman & Bell, Norwood, OH, United States). Briefly, after dewaxing sections in xylene, slides were rinsed in 100% then 95% ethanol. Sections were stained for approximately 16 h with 0.1% Luxol Fast Blue in 95% ethanol and 1:200 glacial acetic acid (Fisher Scientific, Hampton, NH, United States). After staining, sections were rinsed in 95% ethanol and distilled water. Finally, slides were sequentially rinsed in 70% ethanol, distilled water, then 90 and 100% ethanol before being fixed in xylene and cover-slipped (Corning, NY) with DPX mounting medium (Electron Microscopy Sciences, Hatfield, PA, United States). Images were taken on a bright-field BX51 microscope (Olympus, Center Valley, PA, United States) at 10× and 20× magnification.

**TABLE 1:** Information on human post-mortem tissue samples.

Diagnosis	BBID	Age (years)	Sex	PMI (hours)	Cause of death	*ATXN3* CAG repeat sizes
SCA3	1602	48	F	22	SCA3-related	22/73
	1832	49	M	48	SCA3-related	12/73
	816	59	M	NA	SCA3-related	NA/72
	1035	59	F	4	SCA3-related	21/70
	1547	84	F	20	SCA3-related	21/66
Control	1532	43	M	9	Acute perforation and septic shock	NA/NA
	1073	47	M	23	Sudden cardiac arrest	NA/NA
	729	59	M	12	Sudden cardiac arrest	18/21
	14	65	M	24	Acute respiratory distress syndrome	NA/NA
	24	65	M	26	Heart failure	NA/NA
	1432	83	F	21	Renal cell carcinoma	12/19

F, female; M, male; PMI, postmortem interval; NA, not available.

### 2.2. Mice

All animal procedures were conducted in accordance with the United States Public Health Service’s Policy on Humane Care and Use of Laboratory Animals and approved by the University of Michigan Institutional Animal Care and Use of Laboratory Animals. The ATXN3^*Q*82/*Q*6^ Knock-In mouse model (KIQ82), described by Ramani et al. (2017), contains the expanded human CAG repeat domain containing the flanking regions of the *ATXN3* gene inserted into the mouse endogenous gene. The ATXN3^*Q*300/*Q*6^ Knock-In mouse model (KIQ300) recapitulates the aforementioned KIQ82 model but contains a hyperexpanded CAG repeat. Genotyping for both Knock-In mouse models was done using primers flanking the endogenous CAG repeat region (For 5′-TTCACGTTTGAATGTTTCAGG-3′, Rev 5′ -ATAT GAAAGGGGTCCAGGTCG-3′) as previously described (Ramani et al., 2015). The YACQ84 (“Q84”) mouse model, originally derived by Cemal et al. (2002), was genotyped using tail samples collected from pre-weaned animals and confirmed using post-mortem tail samples, as previously described (Moore et al., 2017). *Mobp*-eGFP+ (Gong et al., 2003) (MGI:4847238) genotyping was completed using *Mobp* 5′-GGTTCCTCCCTCACATGCTGTTT-3′ and 5′-TAGCGGCTGAAGCACTGCA-3′ primers. Isolated tail DNA was analyzed for CAG repeat expansion sizes by gene fragmentation analysis (Laragen Inc., Culver City, CA, United States) using *Atxn3* primers (5′-FAM-TTCACGTTTGAATGTTTCAGG-3′ and 5′-ATATGAAAGGGGTCCAGGTCG-3′) for KIQ82 and KIQ300 mice and *ATXN3* primers (5′-FAM-ACAGCAGCAAAAGCAGCAA-3′ and 5′-CCAAGTGCTCCTGAACTGGT-3′) for Q84 mice. Average CAG repeat length for KIQ82 mice was 119.1 ± 6.4, for KIQ300 mice was 297 ± 9.8, and for Q84 mice assessed was 75 ± 2.5. To collect tissue samples for analysis, anesthetized mice were cardiac perfused with 1X PBS solution. For transcriptional and biochemical analyses of age- and sex-matched mice, the left-hemisphere of the dissected brain was macro-dissected into brainstem, cerebellum, spinal cord, and forebrain and flash frozen for RNA, as previously described (Moore et al., 2017). Separate cohorts of Q84 mice were used for behavioral, transcriptional, and histological studies. For Q84;*Mobp*-eGFP+ histological studies, the right hemisphere tissue was post-fixed in 4% paraformaldehyde, sucrose embedded and sectioned for histological analysis, as previously described (Schuster et al., 2022c).

### 2.3. Open field behavioral assessment

Behavioral activity was characterized at 2, 3, 4, 8, and 14 weeks in Q84 mice and control sex- and age-matched littermates (*n* = 16–20 mice/timepoint/genotype). Locomotor activity was evaluated by measuring the number of x/y-axis beam breaks during 30-min trials on a photobeam system open field apparatus (San Diego Instruments, San Diego, CA, United States) as previously described (McLoughlin et al., 2018). Rearing activity was evaluated by measuring the number of z-axis beam breaks during the same trials as previously described (Costa et al., 2013). Weight was recorded at each timepoint prior to behavioral testing. Experimenters were blinded to genotypes during all behavioral tests.

### 2.4. Brain tissue homogenization

Homogenization of macro-dissected mouse brain tissue and human post-mortem brain tissue (obtained from the Michigan Brain Bank, see Table 1 for reference) was completed using a solution of Radioimmunoprecipitation Lysis and Extraction Buffer (ThermoFisher, PI89900, Waltham, MA, United States) and Protease Inhibitor (Roche, 11873580001, Basel, Switzerland), which was added to each tissue sample. Samples were then homogenized using a Next Advance Bullet Blender. The resulting mouse homogenized tissue lysate was used for RNA extraction and human post-mortem tissue lysate was used for Western blot.

### 2.5. RNA isolation and quantitative PCR

RNA was isolated using RNeasy Mini Kits (QIAGEN; Cat # 74004; Germantown, MD, United States), with removal of DNA from RNA isolate using DNAse I kits (QIAGEN; Cat # 79254; Germantown, MD, United States) according to the manufacturer’s protocol. RNA was eluted in RNase-free water and the RNA concentration and purity in isolated samples was determined using an ND-1000 Spectrophotometer. RNA was reverse transcribed using an iScript cDNA synthesis kit according to the manufacturer’s instructions (Bio-Rad, Cat # 1708890; Hercules, CA, United States). Quantitative PCR (qPCR) was performed following the ThermoFisher protocol using TaqMan primers: transcript of interest (FAM) and Beta Actin (VIC) and run on QuantStudio3 qPCR machine using the TaqMan Fast Protocol. TaqMan primers used include the following: *Beta Actin* (ThermoFisher; Cat # Mm02619580; Burlington, MA, United States), *Plp1* (ThermoFisher; Cat #Mm01297210_m1; Burlington, MA, United States), *Ugt8a* (ThermoFisher; Cat #Mm00495930_m1; Burlington, MA, United States), *Mobp* (ThermoFisher; Cat #Mm02745649_m1; Burlington, MA, United States), and *Mal* (ThermoFisher; Cat #Mm01339780_m1; Burlington, MA, United States). Tissue samples from SCA3 mouse models were sex- and age-matched to control wildtype (WT) littermates. Gene expression relative quantification (RQ) was calculated relative to WT/WT samples for each timepoint and normalized to *Beta Actin*, where RQ = 2^–(ddCT)^.

### 2.6. Western blot

Human post-mortem brain tissue lysates were quantified per manufacturer’s instructions for the Pierce BCA Protein Assay Kit (ThermoFisher, Ref # 23227, Waltham, MA, United States). For Western blots, 20 μG of protein lysate was run on 4–20% gradient sodium dodecyl sulfate-polyacrylamide electrophoresis gels (ThermoFisher; XP04200BOX; Waltham, MA, United States) and transferred to a 0.45 μm nitrocellulose membrane (Bio-Rad; Cat # 1620115; Hercules, CA, United States). Membranes were blocked with 5% milk in TBST for 1 h and incubated at 4°C overnight in primary antibodies, including GAPDH (1:10,000; Millipore-Sigma; MAB374; Burlington, MA, United States) and MBP (1:1000; Santa Cruz Biotechnology; sc-66064; Dallas, TX, United States). The next day, membranes were incubated for 1 h with anti-mouse peroxidase-conjugated secondary antibodies (1:5000; Jackson ImmunoResearch Laboratories; West Grove, PA, United States). Protein bands were visualized using the Syngene G:BOX mini imaging system after applying EcoBright Pico HRP (Innovative Solutions; EBPH100; Beverly Hill, MI, United States). Band intensities were quantified using ImageJ analysis software (NIH).

### 2.7. Immunohistochemistry

Phosphate buffered saline perfused brains from 4-, 8-, and 16-week-old Q84;*Mobp*-eGFP+ mice were post-fixed overnight in 4% paraformaldehyde (PFA) and switched to 30% sucrose in 1× phosphate buffered saline (PBS) for long term storage at 4°C. Brains were sectioned and stored in cryostorage as previously described (Moore et al., 2017). Q84;*Mobp*-eGFP+ sections were washed overnight in PBS and again three times for 10 min each the following day. Sections were stained with DAPI (Sigma; D9564; Burlington, MA, United States) for 15 min at room temperature, washed three times with PBS, and mounted with Prolong Gold Antifade Reagent (ThermoFisher; P36930; Waltham, MA, United States). Imaging was performed using a Nikon-A1 Standard Sensitivity confocal microscope with NIS-Elements software. Regions imaged include the basilar pontine nuclei (denoted as pons), deep cerebellar nuclei (DCN), corticospinal tract (CST), corpus callosum (CC), cortex, and striatum. Images were analyzed using CellProfiler software (Jones et al., 2008). Cell counts were obtained by assessing the overlay of *Mobp*-eGFP+ cells and DAPI-stained nuclei in each image, taken as a percentage of total DAPI-stained nuclei, and normalized to the average of WT/WT cell counts.

### 2.8. Statistics

All statistical significance was determined using GraphPad Prism software (La Jolla, CA, United States). Gaussian distribution of qPCR and Western blot data was tested with a Shapiro–Wilk test of normality and significance was assessed utilizing the appropriate parametric (unpaired students *t*-test) or non-parametric (unpaired Mann–Whitney test) statistics. Significance of *Mobp*-eGFP+ cell counts was assessed using a one-way ANOVA with a *post-hoc* Tukey’s multiple comparisons test. Statistical significance for behavioral data was computed using a two-way ANOVA (mixed model) with *post-hoc* Tukey’s multiple comparisons test. Data is reported as the mean ± standard error of the mean (SEM) with all statistical tests set the level of significance at *p* < 0.05.

## 3. Results

### 3.1. Region-specific myelin reduction in end-stage brain tissue from patients with SCA3

**FIGURE 1:**
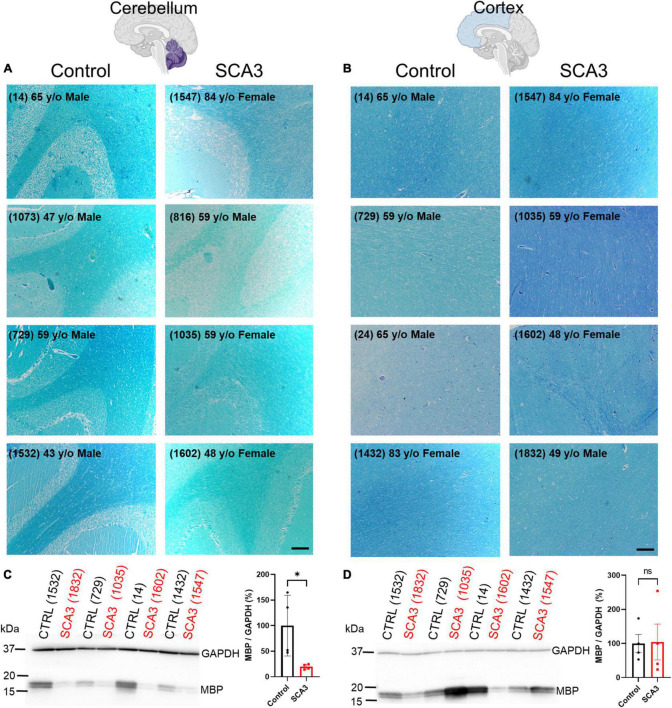
Human post-mortem myelin staining is reduced in the cerebellum and unchanged in the frontal cortex of patients with SCA3 relative to control subject samples. Representative images show post-mortem human **(A)** cerebellum and **(B)** cortex stained with Luxol Fast Blue from controls subjects (left) relative to patients with SCA3 (right). Scale bar 200 μm. Myelin basic protein (MBP) is significantly decreased in the cerebellum of SCA3 patients compared to approximately age-matched controls **(C)**, while MBP varies immensely and is not significantly different between SCA3 and control samples in the frontal cortex **(D)**. Student’s unpaired *t*-test performed after verifying normal distribution of data *via* the Shapiro–Wilk normality test. *p < 0.05.

Similar to many neurodegenerative diseases, it is well-established in SCA3 that certain brain regions are more susceptible to the onset and progression of disease pathology. Identifying vulnerable brain regions in patients has become more accessible with the development of non-invasive, advanced imaging techniques (Lukas et al., 2006; Guimaraes et al., 2013; Kang et al., 2014; Rezende et al., 2018; Piccinin et al., 2020). However, it remains unknown which of these regions is first affected by disease pathology, and what the spatiotemporal spread of disease pathogenesis might be. Our lab recently described oligodendrocyte dysfunction to be one of the earliest and most progressive signatures in the brainstem of a mouse model of SCA3 disease (Schuster et al., 2022c). In addition, analysis of SCA3 post-mortem cerebellar tissue revealed depletion of myelin and mature oligodendrocyte protein compared to controls (Costa et al., 2020; Haas et al., 2021). No previous studies have compared disease-associated oligodendrocyte signatures between affected and unaffected brain regions in SCA3 patient tissue. Therefore, to assess regional mature (myelinating) oligodendrocyte impairments in SCA3 patient tissue, we assessed myelination using a Luxol Fast Blue stain in cerebellar and cortical tissue from SCA3 and control post-mortem subjects (Figures 1A, B and Supplementary Figure 1). Consistent with MRI studies assessing white matter changes (Kang et al., 2014; Arruda et al., 2020; Park et al., 2020; Piccinin et al., 2020; Li et al., 2022), we discovered reduced cerebellar myelin staining in SCA3 patient brains relative to controls (Figure 1A). In contrast, we found similar myelin staining between SCA3 patients and control subjects in the frontal cortex (Figure 1B). In agreement with the Luxol Fast Blue staining, Western blot analysis confirmed significantly lower MBP levels in the cerebellum of SCA3 patients compared to controls (Figure 1C), while MBP in the frontal cortex was unchanged (Figure 1D). This finding corroborates the enhanced vulnerability of the cerebellum to SCA3-related pathology relative to the frontal cortex (Yamada et al., 2001; Kang et al., 2014) and validates regional-specificity of disease-associated oligodendrocyte signatures in human patients (Schuster et al., 2022c). However, because patient post-mortem tissue often only depicts end-stage disease states, it is challenging to determine the progression of myelin loss in affected regions. Mouse models provide the opportunity to analyze pathology at various disease states. In particular, we aimed to characterize the underlying white matter reductions in disease-vulnerable brain regions of SCA3 patients. The failure of oligodendrocytes to differentiate into a mature, myelinating state in a transgenic mouse model of SCA3 (Schuster et al., 2022c) could explain the lower white matter in disease. However, it is unknown whether regional oligodendrocyte maturation impairments are observed across SCA3 mouse models, and if these regional oligodendrocyte impairments relate to disease onset.

### 3.2. SCA3 Knock-In mouse models exhibit regional reduction of oligodendrocyte maturation transcripts

**FIGURE 2:**
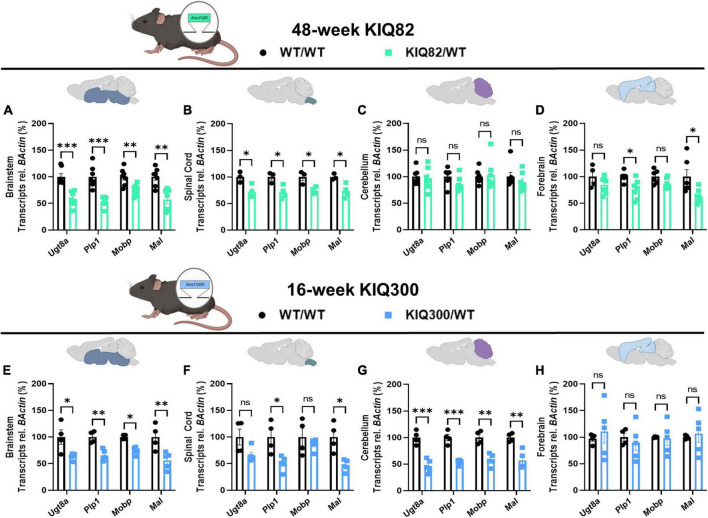
Regional oligodendrocyte transcriptional dysregulation is observed in the Knock-In SCA3 mouse models. QPCR analysis of oligodendrocyte maturation markers in 48-week hemizygous (Q82/WT) KIQ82 mice relative to WT/WT in panel **(A)** brainstem, **(B)** spinal cord, **(C)** cerebellum, and **(D)** forebrain. QPCR analysis of oligodendrocyte maturation markers in 16-week hemizygous (Q300/WT) KIQ300 mice relative to WT/WT in panel **(E)** brainstem, **(F)** spinal cord, **(G)** cerebellum, and **(H)** forebrain. Each data point represents an independent mouse per genotype. Unpaired Student’s *t*-tests or Mann–Whitney tests were performed, depending on normality distribution as determined by Shapiro–Wilk tests: *p < 0.05, ^**^p < 0.01, ^***^p < 0.001, ns, not significant.

To examine differences in susceptibility to oligodendrocyte maturation deficits between brain regions in human-relevant SCA3 mouse models, we quantified transcriptional levels of differentiating and mature oligodendrocyte markers in two Knock-In mouse models that express endogenous levels of the mutant ATXN3 protein (Figure 2). The KIQ82 was originally derived with 82 CAG repeat in the *Atxn3* gene, which lies within the range of patient repeat size (Ramani et al., 2017). At 1 year of age this mouse model did not exhibit deficits on motor behavior tasks including balance beam, rotarod, and open field exploration (Ramani et al., 2017), allowing for assessment of gene changes prior to onset of motor impairment, but after mutant ATXN3 begins to accumulate in nuclei. In the premanifest 48-week-old KIQ82 mouse model, we found four differentiating and mature oligodendrocyte transcripts (*Ugt8a, Plp1, Mobp, Mal*) to be significantly downregulated in the brainstem and spinal cord tissue (Figures 2A, B), whereas no significant gene expression changes among the transcripts assessed were found in cerebellar tissue (Figure 2C). Forebrain samples from these mice showed significant downregulation of *Plp1* and *Mal*, but not *Ugt8a* or *Mobp* (Figure 2D).

In patients with SCA3, the length of the disease CAG repeat in ATXN3 inversely correlates with age of disease onset and directly correlates with disease severity (Maciel et al., 1995; Durr et al., 1996). Therefore, to test the effects of higher CAG repeats numbers, we obtained an earlier onset KIQ300 mouse model, which expresses a hyperexpanded version of the KIQ82 *Atxn3* mutant gene with 300 CAG repeats. The hyperexpanded KIQ300 mice at 16 weeks demonstrated similar patterns of mature oligodendrocyte gene expression downregulated in the brainstem as in the KIQ82 48-week tissue samples, but only significant downregulation of *Plp1* and *Mal* in spinal cord tissue (Figures 2E, F). Interestingly, in this hyperexpanded mouse model, all four differentiating and mature oligodendrocyte transcripts were also significantly downregulated in the cerebellum (Figure 2G). Finally, consistent with patient studies and previous results in these mice, forebrain tissue was the least affected and did not show any significant differences among the mature oligodendrocyte transcripts assessed (Figure 2H). Overall, these results imply regional and temporal differences in transcriptional oligodendrocyte maturation impairments that are exacerbated by endogenous expression of the mutant *ATXN3* gene.

### 3.3. Motor phenotypic onset in SCA3 Q84 transgenic mice occurs concurrently with transcriptional oligodendrocyte maturation dysregulation

**FIGURE 3:**
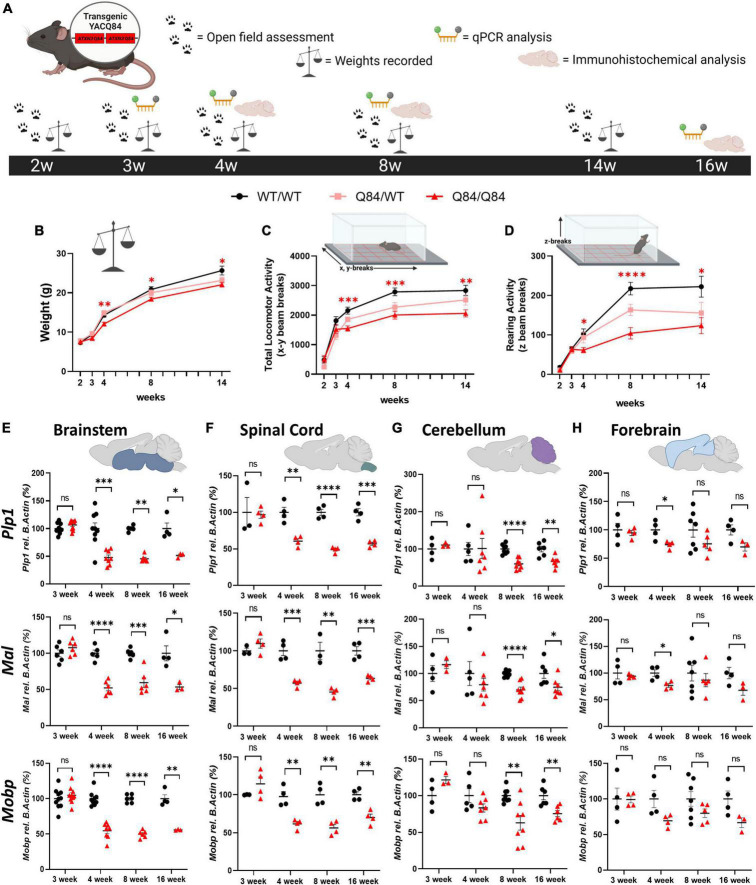
Onset of behavioral deficits in Q84 mice associates with mature oligodendrocyte transcript dysregulation. **(A)** Schematic of the recording timepoints for four phenotypic characterizations of Q84 mice. Tissue was collected for qPCR analysis at 3-, 4-, 8-, and 16-weeks-old. Q84;*Mobp*-eGFP tissue was collected for immunohistochemical analysis at 4-, 8-, and 16-weeks-old. Recordings of panel **(B)** weight, **(C)** total locomotor activity (x-y beam breaks), and **(D)** rearing activity (z beam breaks) in hemizygous (Q84/WT) and homozygous (Q84/Q84) Q84 mice relative to their WT littermates (WT/WT) at 2-, 3-, 4-, 8-, and 14-weeks-old (*n* = 16–20 mice/genotype. Mixed-effects analysis with a *post-hoc* Tukey’s multiple comparison test was performed for behavioral analyses *p < 0.05, ^**^p < 0.01, ^***^p < 0.001, ^****^p < 0.0001; no asterisk, not significant. Red asterisks indicate comparison of Q84/Q84 to WT/WT. **(E,F)** QPCR analysis of oligodendrocyte maturation markers (*Plp1*, *Mal*, *Mobp*) in Q84 mice in the **(E)** brainstem, **(F)** spinal cord, **(G)** cerebellum, and **(H)** forebrain. Each data points represents an independent mouse per genotype. For qPCR analysis, unpaired Student’s *t*-tests or Mann–Whitney tests were performed, depending on normality distribution as determined by Shapiro–Wilk tests: *p < 0.05, ^**^p < 0.01, ^***^p < 0.001, ^****^p < 0.0001; ns, not significant.

With the validation of regional vulnerability to oligodendrocyte dysfunction in both patients with SCA3 and SCA3 Knock-In mouse models, we utilized the Q84 mouse model to further characterize the onset and progression of this disease phenotype through behavioral, transcriptional, and histological analysis (Figure 3A). This mouse model overexpresses the full-length human *ATXN3* gene with a disease-causing expansion of 84 CAG repeats under the endogenous human promoter (Cemal et al., 2002). Our lab has previously published disease-associated oligodendrocyte signatures in the Q84 overexpression mouse model of SCA3 beginning at 4-weeks-old in brainstem regions (Schuster et al., 2022c). Because the earliest behavioral characterization of this Q84/Q84 mouse model, completed at 6 weeks of age, found mice to already be symptomatic in assessed motor tasks (Costa et al., 2013), we sought to define the timepoint at which motor symptoms in this overexpression mouse model begin. To determine the onset and advancement of peripheral disease phenotypes and how they might correspond to oligodendrocyte dysfunction, we first measured motor behavior and weight of Q84/Q84 and Q84/WT mice relative to wildtype littermates at 2, 3, 4, 8, and 14 weeks of age (Figures 3B–D). Longitudinal motor behavior was evaluated *via* Open Field assessment that included both locomotive and exploratory activity. Compared to wildtype littermates, Q84/Q84 mice had similar weights and motor behavior until 4 weeks of age, when significant weight loss and motor deficits became evident and continued through 14-weeks-old (Figures 3B–D).

Interestingly, our motor behavior results are temporally consistent with the onset of disease-associated oligodendrocyte transcriptional signatures previously reported in the brainstem at 4 weeks (Schuster et al., 2022c; Figures 3E, F). We furthered this initial transcriptional characterization by assessing the temporal progression of mature oligodendrocyte transcript disruption across vulnerable and non-vulnerable SCA3 brain regions. Using three prominent mature oligodendrocyte markers (*Plp1*, *Mal*, and *Mobp*), we distinguished temporal vulnerability between brainstem, spinal cord, cerebellar, and forebrain tissue in Q84/Q84 mice relative to wildtype littermates (Figures 3E–H). We found that mature oligodendrocyte transcripts in the brainstem and spinal cord were the first to be dysregulated, beginning between 3 and 4 weeks of age and continuing through 16 weeks (Figures 3E, F). This was followed by downregulation of mature oligodendrocyte markers in cerebellar tissue that was significant by 8-weeks-old and continued through 16 weeks (Figure 3G). It is important to note that these results represent whole cerebellum analysis, which could be diluting the effects on the most severely affected region within the cerebellum, the DCN. Finally, we saw little to no significant changes in mature oligodendrocyte transcripts in the forebrain at all timepoints assessed, with the exception of *Plp1* and *Mal* at 4 weeks of age (Figure 3H). These results indicate a temporal progression of mature oligodendrocyte transcriptional downregulation across brain regions in which the brainstem and spinal cord are the most vulnerable, followed by the cerebellum. The forebrain appears to be a less vulnerable brain region in Q84/Q84 mice through 16 weeks, which is consistent with SCA3 KI mouse data (Figure 2), post-mortem human samples (Figure 1), and patient literature describing substantially less cortex atrophy at late-stage and end-stage disease compared to white matter regions (D’Abreu et al., 2012; Rezende et al., 2018).

### 3.4. Mature oligodendrocytes are spatiotemporally reduced in SCA3 vulnerable brain regions

**FIGURE 4:**
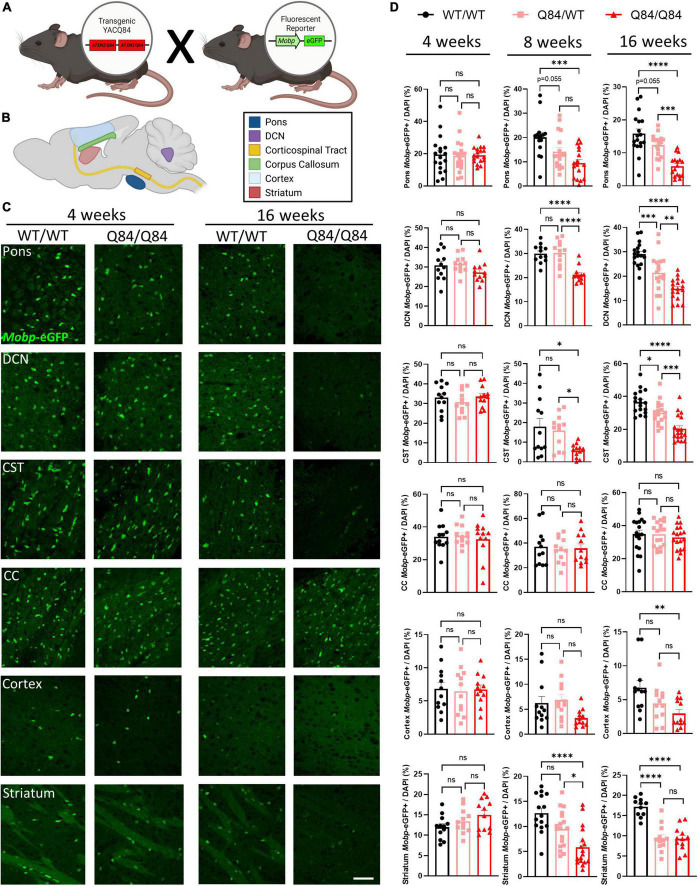
Mature oligodendrocytes are reduced in a spatiotemporal pattern that corresponds to disease progression. **(A)** Mouse cross between Q84 SCA3 mice and *Mobp*-eGFP reporter mice. **(B)** Depiction of where images were taken for histologically assessed regions. **(C)** Representative images of *Mobp*-eGFP+ mature oligodendrocytes in the pons, DCN, CST, CC, cortex, and striatum at 4 weeks (left) and 16 weeks of age (right). Scale bar 50 μm. **(D)** Quantification of mature oligodendrocytes in the pons, DCN, CST, CC, cortex, and striatum of Q84;*Mobp*-eGFP+ mice at 4 (left), 8 (middle), and 16 weeks of age (right) (*n* = 4–6 mice/genotype, 3 images/mouse). One-way ANOVA with a *post-hoc* Tukey’s multiple comparisons test was performed: *p < 0.05, ^**^p < 0.01, ^***^p < 0.001, ^****^p < 0.0001; ns, not significant.

We have previously demonstrated that mature oligodendrocytes are decreased in SCA3 vulnerable brains regions, including the pons, DCN, and CST, and that in a non-vulnerable region, the corpus callosum, mature oligodendrocyte counts remain unchanged in symptomatic Q84/Q84 mice (Schuster et al., 2022c). This study, however, did not explore the temporal onset and progression of this maturation deficit. To understand the longitudinal cellular effects of oligodendrocyte maturation transcript dysregulation in a region-specific manner, we histologically analyzed mature oligodendrocyte cell counts in Q84 mice in both vulnerable and non-vulnerable brain regions relative to their wildtype littermates (Figure 4). By crossing Q84 mice with *Mobp*-eGFP reporter mice (Figure 4A), we were able to visualize mature oligodendrocytes and further assess the spatiotemporal effects of oligodendrocyte maturation impairments at 4, 8, and 16 weeks of age in the pons, DCN, corticospinal tract (CST), corpus callosum (CC), cortex, and striatum (Figures 4B–D).

**FIGURE 5:**
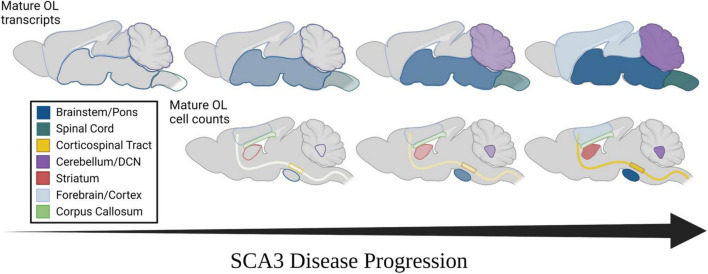
Spatiotemporal progression of mature oligodendrocyte transcript dysregulation and cell count reduction in mouse models of SCA3. **(Top)** Temporal progression of regional transcriptional downregulation of mature oligodendrocyte markers as observed in mouse models. **(Bottom)** Temporal reduction of regional mature oligodendrocyte cell counts as observed in mouse models. Horizontal arrow represents disease progression over time. Transparency and shade of regional colors represent magnitude of oligodendrocyte maturation impairments (darker/less transparent = more affected, lighter/more transparent = less affected).

At 4 weeks of age, the timepoint at which transcriptional dysregulation and behavioral deficits begin, we found that differences in mature oligodendrocyte cell counts are not yet evident in any brain region assessed (Figures 4C, D, left). This is intriguing as the highest rate of myelination in mice occurs between 1 and 5 weeks of age (Spitzer et al., 2019), indicating that the reduction of mature oligodendrocytes in SCA3 disease was likely not a result of developmental distress. By 8-weeks-old, mature oligodendrocyte counts in Q84/Q84 mice relative to wildtype littermates were reduced 30–50% in the most vulnerable SCA3 brain regions of the DCN and pons, respectively (Figure 4D, middle). As previously denoted, while whole cerebellar transcripts did not change until later stages of disease, the DCN is significantly affected early in disease progression. Further histological evaluation demonstrated the regional specificity of oligodendrocyte maturation impairments extended to both the CST and striatum and temporally matched the onset of dysfunction in the pons and DCN (Figure 4D, middle). This is consistent with patient imaging data showing similar atrophy between vulnerable regions (Klaes et al., 2016; Inada et al., 2020). Mature oligodendrocytes in the corpus callosum and cortex, however, remain unchanged at this timepoint (Figure 4D, middle). Analyzing 8 weeks later, the affected brain regions, including the pons, DCN, CST, and striatum, in Q84/Q84 mice showed a reduction of nearly half the mature oligodendrocytes compared to wildtype littermates at 16 weeks of age (Figures 4C, D, right). The majority of these vulnerable regions also displayed a disease dose-dependent reduction in mature oligodendrocyte cell counts. By this time point, onset of impaired oligodendrocyte maturation became clear in the cortex, as well, though not as significantly (Figures 4C, D, right). A lack of transcriptional changes at 16 weeks in the corresponding forebrain region could be due to the higher degree of regional specificity of histological assessments since oligodendrocyte count analysis was restricted to the motor cortex. Even though by 16 weeks the majority of disease phenotypes are evident in Q84 mice, the corpus callosum still showed no changes in mature oligodendrocyte counts, suggesting this white matter tract may be preserved in SCA3 pathogenesis. Importantly, prior work demonstrated that while mature oligodendrocyte cell counts were decreased in vulnerable brain regions, total oligodendrocyte lineage cell counts remained unchanged, suggesting this is a maturation impairment rather than an increase in cell death (Schuster et al., 2022c). When assessing the cell-autonomous dysfunction of primary SCA3 oligodendrocytes, we found no differences in total cell counts, indicating disease oligodendrocytes are not dying at a higher rate than wildtype cells (Schuster et al., 2022a). Moreover, there were no changes in oligodendrocytes expressing the proliferation marker Ki-67, suggesting that there is no compensation for cell death (Schuster et al., 2022a). Therefore, rather than the reduction of mature oligodendrocytes being a cause of increased cellular death, these data portray an inability of SCA3 oligodendrocytes to fully mature into myelinating cells. In combination, these results depict spatiotemporal reduction of oligodendrocyte maturation that align with regional vulnerability of SCA3 pathogenesis (Figure 5).

## 4. Discussion

Spinocerebellar ataxia type 3 (SCA3) research has historically focused on the dysfunction of neuronal cells in disease pathogenesis, despite patient data showing early atrophy of white matter regions (Joers et al., 2018; Rezende et al., 2018; McLoughlin et al., 2020; Li et al., 2022). In-life assessments *via* MRI and DTI reveal that tissue degeneration in the spinal cord, cerebellar peduncles, and substantia nigra occurs early in disease pathogenesis, whereas the cerebral cortex is not affected until later stages of disease (Rezende et al., 2018; Piccinin et al., 2020; Wan et al., 2020). Regional deterioration has been reported in several other neurodegenerative diseases (Scahill et al., 2002; Hobbs et al., 2010; Zhang et al., 2016; Wijeratne et al., 2021; Pieperhoff et al., 2022), many of which also demonstrate white matter abnormalities. The basis and progression of this regional vulnerability, however, has been relatively unexplored. We previously reported that transcripts involved in the maturation of oligodendrocytes, the primary cells found within white matter, are dysregulated early in an SCA3-vulnerable brain region through a toxic gain-of-function mechanism (Schuster et al., 2022a,c). Here, we extend our foundational studies to relate SCA3 phenotypic and regional vulnerability to the onset and progression of disease-associated oligodendrocyte signatures in both Knock-In and overexpression mouse models of SCA3 (Figure 5). In line with SCA3 patient imaging of white matter changes (Kang et al., 2014; Li et al., 2022), we observed transcriptional dysregulation of oligodendrocyte maturation in SCA3 mice first in brain regions most vulnerable to SCA3, including the brainstem and spinal cord, followed by the cerebellum, with little to no changes in the forebrain. More detailed regional histological assessment revealed the SCA3 vulnerable DCN within the cerebellum is in fact similarly affected by oligodendrocyte maturation impairments as the pons. This is also true for the CST and striatum, though the motor cortex only shows reduced cells at later stages of disease and the corpus callosum shows no effects at any timepoint. Our work highlights the utility of SCA3 mice as a paradigmatic model of neurodegenerative disease for further exploration of the potential relationship between this progressive non-neuronal disease phenotype and regional vulnerability.

Because SCA3 is a monogenic neurodegenerative disease, the onset and progression of disease phenotypes are dependent upon both the CAG repeat size and the number of mutant *ATXN3* alleles. In patients, studies have shown that higher repeat sizes correlate with earlier onset of disease (Maciel et al., 1995; Durr et al., 1996; Jardim et al., 2001; Costa and Paulson, 2012) and that age of disease onset for homozygous patients is earlier than heterozygous patients (Lang et al., 1994; Lerer et al., 1996; Carvalho et al., 2008; Shang et al., 2018; Li et al., 2020). It is therefore imperative to study the dosage effects of *ATXN3* to fully understand SCA3 pathogenesis. Both of these *ATXN3*-dependent phenomena seen in patients have been recapitulated in SCA3 mouse models, and we demonstrate here that we also see this manifest with disease-associated oligodendrocyte signatures. Similar to SCA3 patient reports, Knock-In mice with a greater number of CAG repeats have earlier dysfunction than Knock-In mice with fewer repeats. Specifically, mice with 300 CAG repeats (KIQ300) show regional oligodendrocyte maturation transcription impairment at an earlier age than KIQ82 mice. Additionally, disease gene dosage plays a role in disease-associated oligodendrocyte signatures, as we see dose-dependent maturation deficits in Q84 mice that overexpress two human mutant *ATXN3* genes at hemizygosity (Q84/WT) and four mutant genes at homozygosity (Q84/Q84). We further show similar timing of oligodendrocyte maturation impairments in mice with high repeat number (KIQ300 mice) and high gene dosage (Q84/Q84), in that both have significant transcriptional downregulation of mature oligodendrocyte transcripts at 16 weeks of age. Occurring later or to a lesser extent is dysfunction in mice with lower gene dosage (Q84/WT) and repeat size (KIQ82 mice). These results establish a link between disease-associated oligodendrocyte signatures, *ATXN3* gene dosage, and number of CAG repeats.

*ATXN3*-dependent phenotypes can be observed in behavioral assays, as well (Costa et al., 2013). The results presented here extend the behavioral characterization of the Q84 SCA3 mouse model and associate motor impairment with oligodendrocyte dysfunction onset. We report concurrent pathogenesis of behavioral phenotypes in Q84 mice with mature oligodendrocyte transcript downregulation in the brainstem and spinal cord that occurred between 3 and 4 weeks of age. As Q84 mice are behaviorally and transcriptionally indistinguishable from wildtype mice at three-weeks-old, yet motor phenotypes and regional oligodendrocyte transcriptional dysregulation begin at 4 weeks, these disease features are unlikely to derive from a developmental deficit. As motor impairment progresses in the Q84 mice, so too does the severity of disease-associated oligodendrocyte signatures. This highlights the potential systematic importance of the brainstem and spinal cord in locomotor activity. The regions that are the first to display SCA3 pathology in these mice could be targeted for spatial manipulations to study behavioral manifestations of cellular and molecular disease-associated changes. Emphasizing the relationship between motor activity and oligodendrocyte dysfunction, a recent study from our lab demonstrated that the gene silencing of *ATXN3* with an antisense oligonucleotide rescues behavior impairment and recovers oligodendrocyte maturation impairments in Q84 mice (Schuster et al., 2022b). Future studies should explore the association of behavioral phenotype onset with spatiotemporal mature oligodendrocyte transcript changes in the Q82 Knock-In mouse model, as well as the hyperexpanded Q300 model, to determine if this is consistent at endogenous expression levels.

Another approach to investigating regional vulnerability is to instead address regional protection. We can ask what contributes to the protection of certain brain regions that are affected later in disease, such as the cortex or corpus callosum. Because many of the vulnerable regions in SCA3 are made up of myelin-rich white matter, it is intriguing that the corpus callosum, as one of the most prominent white matter tracts in the brain, is not affected. We recently demonstrated that in symptomatic Q84 mice, the corpus callosum is also resistant to myelination abnormalities, and that treatment with an anti-*ATXN3* antisense oligonucleotide led to increased myelin sheath thickness, or hypermyelination (Schuster et al., 2022b). This suggests less vulnerable, or “protected” brain regions may engage a protective mechanism to prevent oligodendrocyte loss and myelin thinning in disease. It is also possible oligodendrocytes in different regions are inherently different, and certain subpopulations are more vulnerable to the effects of disease. Indeed, oligodendrocytes have been shown to be transcriptionally, functionally, and spatially heterogeneous, and to respond to aging and disease in diverse ways (Crawford et al., 2016; Falcao et al., 2018; Marques et al., 2018; Spitzer et al., 2019; Floriddia et al., 2020; Marisca et al., 2020). To be able to untangle the regional effects of oligodendrocyte vulnerability or protection in SCA3, future work will need to comparatively investigate both types of brain regions. These studies will not only inform the spatiotemporal progression of SCA3 oligodendrocyte maturation impairments, but can also be used as the foundation for exploring regional effects of several other neurodegenerative disease with oligodendrocyte signatures.

Besides ATXN3 nuclear accumulation, few cellular pathologies have been shown to track with SCA3 disease progression. We have demonstrated the onset and progression of disease-associated oligodendrocyte signatures parallel those of disease phenotypes. The determination of these timestamps of dysfunction allows for future studies to investigate the inherent differences between brain regions that make some vulnerable to disease. To do this, we can draw from findings in other neurodegenerative diseases. Regional vulnerability is observed across the spectrum of neurodegenerative diseases including the hippocampus in Alzheimer’s disease, the basal ganglia and neocortex in Huntington’s disease and the substantia nigra in Parkinson’s disease (Fu et al., 2018). Uncovering what underlies regional vulnerability in these neurodegenerative diseases will improve our understanding of the pathophysiology of these diseases and possible mechanisms for therapies. Toward SCA3 in particular, future work should leverage a more powerful method of transcript analysis such as single-cell RNA sequencing or spatial transcriptomics to better elucidate genome-wide changes across different cell types and between specific areas within a brain region, such as the DCN and lobules in the cerebellum. In sum, our results establish the significance of oligodendrocyte maturation dysfunction in SCA3 as a paradigmatic neurodegenerative disease across three SCA3 mouse models and patient post-mortem brain tissue, establishing timepoints at which oligodendrocytes could be a potential target for therapeutic intervention. This work reaffirms the importance of non-neuronal cells and provides further impetus to elucidate the causal or correlational role of oligodendrocytes in SCA3 pathogenesis.

## Data availability statement

The original contributions presented in this study are included in the article/Supplementary material, further inquiries can be directed to the corresponding author.

## Ethics statement

Animal studies were reviewed and approved by the University of Michigan Institutional Animal Care and Use Committee (IACUC). All animal procedures were conducted in accordance with the United States Public Health Service’s Policy on Humane Care and Use of Laboratory Animals and approved by the University of Michigan Institutional Animal Care and Use of Laboratory Animals.

## Author contributions

HM, KS, and DD conceptualized and designed the study. KS, DD, AP, JM, SJ, NS, VS, and HM acquired and analyzed the data. KS, AP, DD, JM, SJ, NS, VS, and HM drafted/edited the text and prepared the figures. All authors contributed to the article and approved the submitted version.
