# Supplementary material for: Disease-associated oligodendrocyte signatures are spatiotemporally dysregulated in spinocerebellar ataxia type 3
Source: Front Neurosci. 2023 Feb 15;17:1118429. doi: 10.3389/fnins.2023.1118429 (PMC9975394; doi:10.3389/fnins.2023.1118429)
Supplement: Supplementary file 1 [file Image_1.pdf]

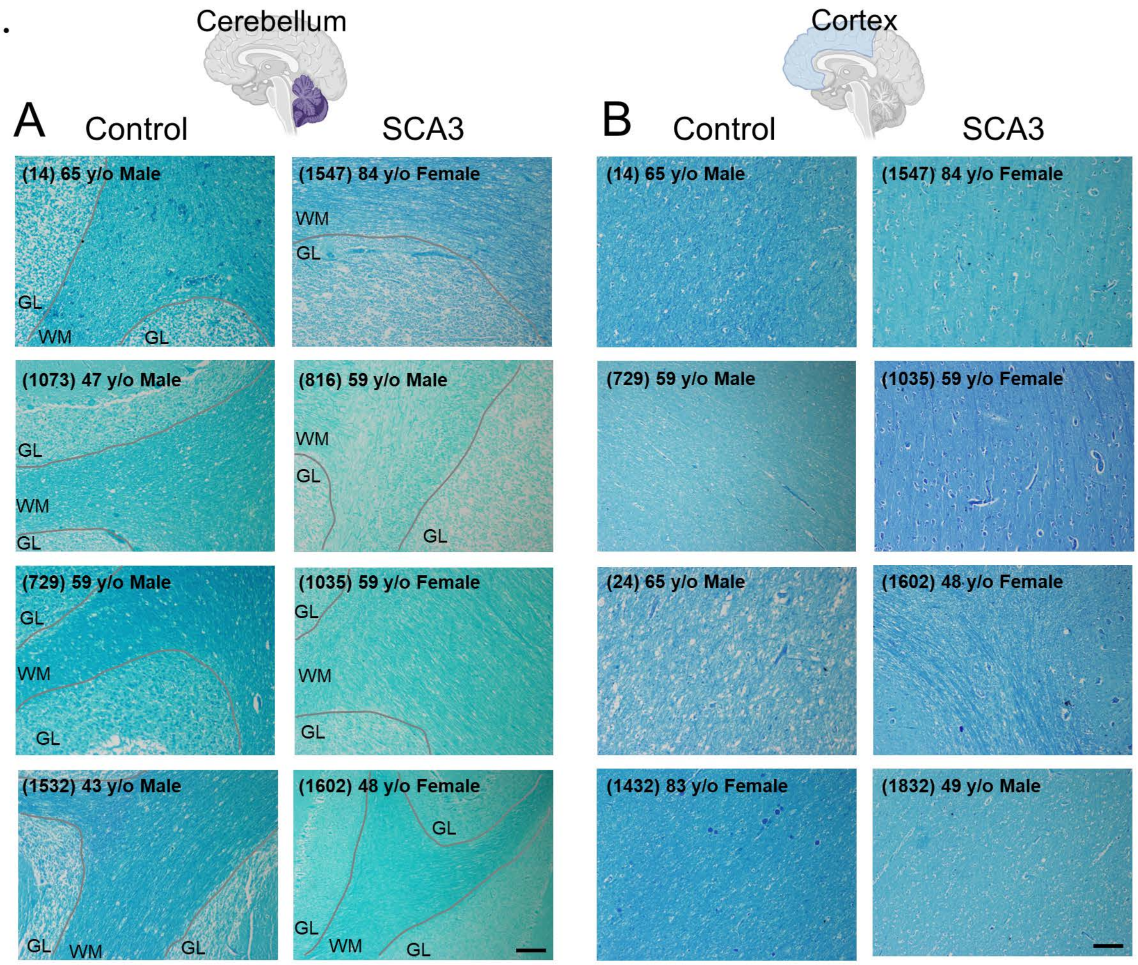

Supplementary Figure 1. High magnification (20x) images of luxol fast blue staining in human cerebellum (A) and frontal cortex (B) of patients with SCA3 and approximately age-matched controls. For clarity, gray lines in cerebellum tissue delineate white matter (WM) from granular layers (GL). Scale bar 100 µm.
